# Experiment and simulation study of the effect of ethanol and compound additives on the urea-based selective non-catalytic reduction process under moderate temperature conditions

**DOI:** 10.1098/rsos.180969

**Published:** 2018-10-24

**Authors:** Bang Wu, Ge Pu, Jiantai Du

**Affiliations:** Key Laboratory of Low-grade Energy Utilization Technologies and Systems, Chongqing University, Ministry of Education of PRC, 400030, People's Republic of China

**Keywords:** compound additives, selective non-catalytic reduction, synergistic effect, reaction routes, NO_X_OUT

## Abstract

An experiment and simulation study of the effect of using liquid additives on the selective non-catalytic reduction (SNCR) process is presented, providing a novel way for plants reducing NO_X_ emissions. An experimental study is conducted in an entrained flow reactor, and CHEMKIN is applied for simulation study. Ethanol additive can effectively shift the temperature window of the NO_X_OUT process to a lower range and the NO_X_OUT efficiency ranges from 29 to 56% at 700–800°C. Furthermore, ethanol additive has a significant inhibitory effect on ammonia slip. Na_2_SO_4_ and C_2_H_5_OH can be combined into a compound additive, which has a synergistic effect on NO reduction. The addition of methanol can greatly promote denitrification efficiency from 650°C to 725°C, indicating the potential of compound additives in NO reduction. The HNCO + OH = H_2_O + NCO pathway is also proven to be enhanced for ethanol decomposition, thereby providing OH•, which is active in NO reduction. Finally, the reaction routes for ethanol on the urea-based SNCR process at the proper temperature are proposed.

## 1. Introduction

Among the causes of current environmental issues, nitrogen oxide (NO_X_) emission plays an important role and leads to photochemical pollution, acid rain and particulate matter. Several technologies have been developed to reduce NO_X_ emissions, which involve controlling the emission before, during and after the combustion process [1]. The main denitrification technologies are selective non-catalytic reduction (SNCR) technology and selective catalytic reduction technology [2]. SNCR technology is considered a promising option for NO_X_ reduction because it does not require an expensive catalytic and because of its good removal efficiency. Thus, it is widely adopted in industries. SNCR processes have three main types according to reductants: thermal De-NO_X_, NO_X_OUT and RAPRENO_X_ [3]. However, to ensure effective NO_X_ removal, the SNCR process is always limited by a narrow temperature range of 850–1100°C. Below this limit, the reduction process occurs too slowly to effectively reduce NO_X_, and ammonia slip may occur; at higher temperatures, the oxidation of reductants may lead to NO_X_ formation rather than NO_X_ reduction. Fortunately, this restricted narrow temperature window could be widened and dropped to a lower temperature range through the injection of effective additives together with reductants, although gaseous additives cannot be transported and stored properly because they have a risk of exploding. Many scenarios are characterized with NO_X_-polluted flue gas at low temperatures in practice, such as flue gas from glass kilns. Therefore, a suitable additive needs to be found to shift the temperature window to moderate temperature conditions.

Various additives have been investigated to widen the temperature window and enhance NO_X_ reduction efficiency. Carbonaceous gases, such as CO and alkanes, were reported to have a great promotion effect on NO_X_ reduction. CO can be used to shift the temperature window to a lower range without affecting the maximum NO_X_ removal efficiency [4]. Similar results were obtained for shifting the temperature window to a lower range, but CO would decrease the NO_X_ removal efficiency [5]. CH_4_ is another suggested alternative because it has a more positive effect on the SNCR process compared with CO [6]. Given some limitations of gas additives, the performance of alkali metal additives in NO reduction was investigated. Results showed that Na/K additives had a certain promoting effect on NO removal, following the sequence of NaCl > NaOH ≈ Na_2_CO_3_ > KCl [7]. Other research indicated that at a temperature of 700°C, NO removal efficiency was 43.86–60.76% in the SNCR process with Na/K additives [8]. Alcohols and some volatile organic compounds were also provided for the SNCR process. Experimental results proved that they induce a downward shift up to more than 100 K of the optimal temperature window [9].

The chemical mechanisms of the SNCR process have also attracted research attention. CO + OH → CO_2_ + H was regarded as a main reason CO shifted the optimal temperature window to a lower range, as indicated by simulation analysis [10]. NO–NH_3_, NO_2_–NH_3_ and O_2_–NH_3_ reaction models were established to explore the effect of Gibbs free energy, reaction rate constant and reaction temperature on the reaction rate [11]. A new approach to injecting Na_2_CO_3_ additive was found: NaO + H_2_O = NaOH, NaOH + O_2_ = NaO_2_ + OH, and NaOH + M = Na + M + OH, which could generate more OH• and NH_2_, promoting NO reduction [12]. Fe elements were also found in the mechanism Fe^2+^ + NH_2_ + NO → Fe^3+^ + N_2_ + H_2_O, which directly participated in the NO removal process [13]. Chemical mechanisms models are mainly used to briefly describe the role of individual additives with parametrized models. Accurate interpretations of the influence of the additives remain few.

The primary objective of this research is to contribute to a better understanding of the effects of liquid additives on the urea-based SNCR process in moderate temperature conditions, thereby contributing to the reduction of NO_X_ emissions from plants. Experimental investigations are conducted in a tubular reactor with simulated flue gas, and sensitivity analysis is adopted to identify the important elementary reactions. The final NO_X_OUT chemical reaction routes are suggested for the ethanol additive on the urea-based SNCR process.

## 2. Material and methods

### 2.1. Materials

Pure urea (CO[NH_2_]_2_ content higher than 99%), absolute ethanol, sodium sulfate, absolute methanol and deionized water were used to prepare the reducing reagents. The tested additives are listed in table 1.

**Table 1.** Additives adopted in the experiments.

additives	composition
C_2_H_5_OH	2.5% urea + C_2_H_5_OH
Na_2_SO_4_	1% urea + C_2_H_5_OH + Na_2_SO_4_
CH_3_OH	1% urea + C_2_H_5_OH + CH_3_OH + Na_2_SO_4_

### 2.2. Experimental set-up and procedure

**Figure 1.**
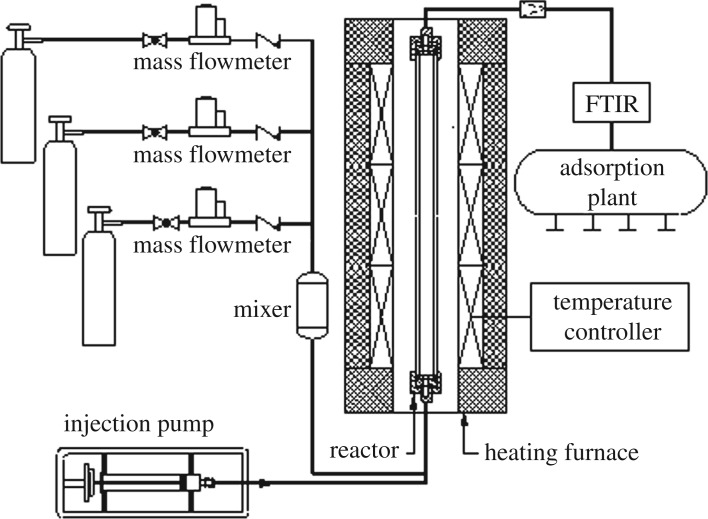
Schematic of the entrained flow reactor for SNCR process.

Experiments were conducted to study the influences of liquid additives on urea-based SNCR process. These experiments were performed in an entrained flow reactor with simulated flue gas, as illustrated in figure 1, which mainly included a gas supplied system, an injection pump, an electric-heated furnace and a measurement system. The reactor consisted of a 1 m long tube with an inner diameter of 26 mm, which served as the SNCR reaction zone and was held in a vertical position. Three electric heaters were located along the tube to keep the SNCR reaction temperature in the 650–900°C range by an automatic temperature controller, and the middle section of the tube with a constant temperature was located at 20–80 cm which signified the length of the isothermal zone is about 60 cm. Both ends of the tube were insulated by insulating cotton and the residence time of the simulated gas was 1.2 s in the isothermal region dependent on the temperature because the mass flow was held constant, guaranteeing the SNCR reactions execute completely.

Simulated flue gas containing 3% O_2_ and 410 ppm NO was mixed with balance N_2_ in a gas compartment. The total flow rate of simulated flue gas was maintained at 2.5 l min^–1^. The reducing agent solutions (urea, additives) were injected into the SNCR reaction zone by a syringe pump. The nozzle was located at the bottom part of the tubular reactor, and N_2_ was used as a carrier gas to increase the momentum of the solutions and for mixing within the simulated flue gas. The quantities of N_2_ and other gas were controlled by mass flow controllers accurately according to the normalized stoichiometric ratio of N-agent to NO (NSR) and a certain additive to NO, respectively.

A drying tube that contained anhydrous calcium chloride was employed to remove water. Then, sample gas components, such as NO, N_2_O, NO_2_, CO_2_, NH_3_, CO and other hydrocarbons were continuously measured by using an Fourier transform infrared (FTIR) flue gas analyzer (ProtIR 204 M), whose measurement range was from 0 to 1000 ppm. The accuracy of the FTIR reached a maximum of ±1% because of some uncertainties, such as spectral interferences and the calibration procedure in the FTIR measurements. Afterwards, the remaining gas was treated with an absorbent to prevent effects on the environment and human health.

*η*_NO_, the NO removal efficiencies of the SNCR process, were calculated as follows:

2.1 $$\eta_{\mathrm{NO}}=\left(\frac{1-C_{\mathrm{NO},\mathrm{out}}}{C_{\mathrm{NO},\mathrm{in}}}\right)\times 100\text{\%},$$

where *C*_NO,in_ and *C*_NO,out_ is the inlet and outlet NO concentration of the SNCR process, ppm, respectively.

### 2.3. Mechanism modelling

In this research, 80 species and 374 reactions (listed in the electronic supplementary material) are reified in the model to simulate the performance of SNCR reaction with additives. CHEMKIN is used for the simulation and the code of plug flow reactor is adopted not only to model the SNCR process but also to perform sensitivity analysis.

The sensitivity is defined as [14]

2.2 $$S_{i}=\frac{k_{i}}{F(k_{i})}\frac{\partial F(k_{i})}{\partial (k_{i})}=\frac{\partial \ln(F(k_{i}))}{\partial \ln(k_{i})},$$

where *S_i_* stands for the local sensitivity coefficient of the *i* reaction to the target function *F*, which is the NO concentration and *k_i_* is the rate coefficient of the *i* reaction.

## 3. Results and discussion

### 3.1. Effect of ethanol on the urea-based SNCR process

**Figure 2.**
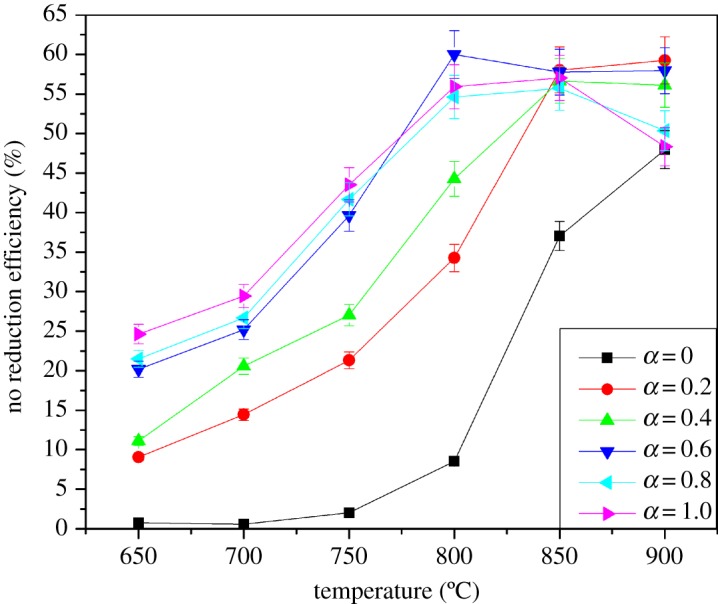
Effect of ethanol on the urea-based SNCR process.

The symbol *α* is defined as the molar ratio of additives to NO. The effect of ethanol on NO reduction during the SNCR process when *α* = 0, 0.2, 0.4, 0.6, 0.8, 1.0 is shown in figure 2. Pure urea shows little NO removal efficiency at the range of 650–800°C. The best NO removal efficiency is less than 10%, although the efficiency increases rapidly after 800°C. Urea may have completed the decomposition at around 550°C, generating equal amounts of NH_3_ and HNCO, but the limited conversion of NH_3_ to NH_2_ could be responsible for the poor NO removal performance [15]. Interestingly, the efficiency of NO reduction improved significantly during the moderate temperature with ethanol addition as *α* varied from 0.2 to 1.0.

Evidently, the NO reduction efficiency of urea-C_2_H_5_OH solution was slightly lower at 750°C and was less than 40%, but the SNCR efficiency of urea-C_2_H_5_OH solution increased gradually with the increase in the amount of ethanol. When *α* = 0.6, the amount of ethanol was significant to the promotion of NO removal, whose efficiency reaches 60%. After 800°C, the oxidation of ethanol became more complete to form CO_2_ and H_2_O instead of OH• so that the SNCR efficiency of urea-C_2_H_5_OH solution showed a decreasing trend as the temperature increased. The NO route (1)–(6) overtaking the NO removing reactions (7)–(8) at high temperature may cause reduction efficiency to fall [16]. *α* = 0.6 is regarded as the optimal choice for adding ethanol in the urea-based SNCR process, although ethanol enhances NO removal when *α* = 0.6, 0.8, 1.0, considering the minimal increase and the economic cost.

$$\begin{array}{rl} & NH_{2}+\mathrm{OH}=\mathrm{NH}+H_{2}O\;\left(1\right)\\ & \mathrm{NH}+O_{2}=\mathrm{HNO}+O\;\left(2\right)\\ & \mathrm{NH}+\mathrm{OH}=\mathrm{HNO}+H\;\left(3\right)\\ & NH_{2}+\mathrm{HNO}=NH_{3}+\mathrm{NO}\;\left(4\right)\\ & \mathrm{HNO}+O=\mathrm{NO}+\mathrm{OH}\;\left(5\right)\\ & \mathrm{HNO}+M=H+\mathrm{NO}+M\;\left(6\right)\\ & NH_{2}+\mathrm{NO}=N_{2}+H_{2}O\;\left(7\right)\\ & NH_{2}+\mathrm{NO}=\mathrm{NNH}+\mathrm{OH}\;\left(8\right) \end{array}$$

### 3.2. Effect of NSR on the urea-C_2_H_5_OH-based SNCR process

NSR represents the mole ratio of urea to NO, which is normally controlled between 1.0 and 2.0 to ensure good NO reduction [17]. It is a key factor in SNCR application, which not only relates to the NO reduction efficiency but also determines the ammonia slip and operation cost.

**Figure 3.**
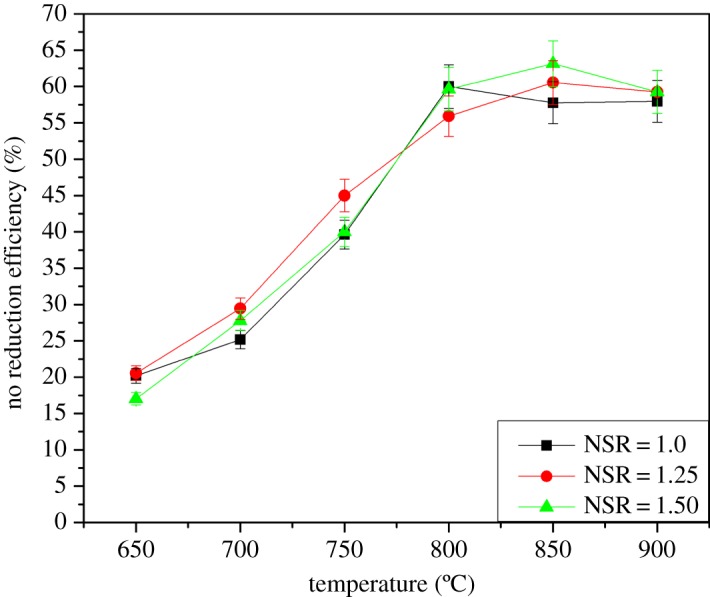
Effect of NSR on the urea-C_2_H_5_OH-based SNCR process.

Figure 3 depicts the effect of NSR on the urea-C_2_H_5_OH-based SNCR process. Three different values of NSR are examined, and the efficiency shows a maximum increase when NSR = 1.25 below 750°C, in which NO reduction increases from 29 to 56% at the range of 700–800°C. The increase is greater than the other two curves when NSR = 1.5 because the temperature is higher than 800°C. This finding indicates that the increase in NSR has a certain effect on NO reduction. However, the impact is negligible under a high temperature (approx. 900°C). To a certain degree, the results are in agreement with those of Zhou, who concluded that the increase in NSR leads to the increase in NH_2_ and NCO, thereby explaining the promotion of NO reduction [18]. When the reduction reaction tends to saturation, continuing to increase NSR is not advisable. As NSR increases, so does the tendency of NH_3_ to slip. Thus, NSR = 1.25 is a good choice for urea-C_2_H_5_OH solution removing NO.

### 3.3. Effect of urea-C_2_H_5_OH on secondary pollutants

**Figure 4.**
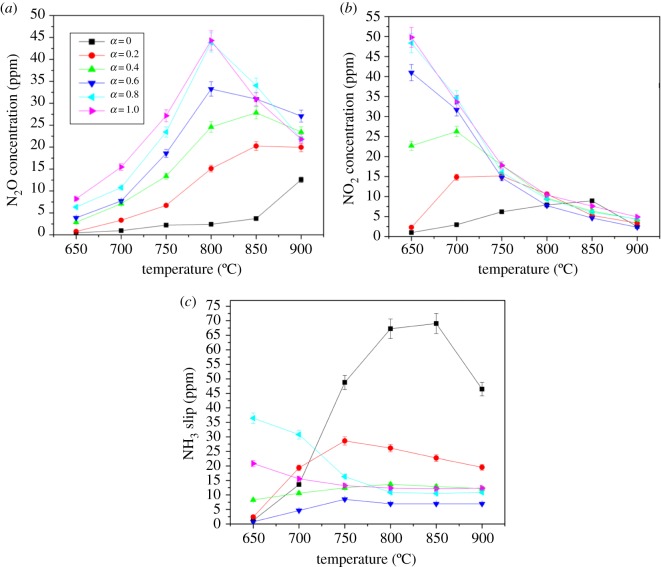
Effect of urea-C_2_H_5_OH on secondary pollutants.

Enhancing NO removal is the goal. However, the emission of secondary pollutants should not be ignored. As shown in figure 4*a*, the addition of ethanol increases the emission level of N_2_O. As the ethanol concentration increases, the peak value of N_2_O emission shifts from the optimal reaction temperature to a lower temperature, and the value increases obviously, thereby indicating that ethanol can slightly favour N_2_O emissions in moderate temperature conditions. Moreover, the increase is positively correlated with the addition. When *α* = 0.6, the maximum emission of N_2_O reaches 33 ppm at 800°C, while continuously adding ethanol has little impact on the emission of N_2_O. As for NO_2_, shown in figure 4*b*, adding ethanol to some extent increases the emission of NO_2_ and increases with continued addition. When *α* = 1.0, the NO_2_ emission is up to 50 ppm at 650°C, but the above conditions reflect just below 750°C; the addition of ethanol makes little sense to NO_2_ formation after 800°C. The oxidation of NO into NO_2_ at a moderate temperature during the SNCR process may explain the relatively high concentration of NO_2_. Above 800°C, a large amount of NO_2_ converts into N_2_, causing the NO_2_ concentration to fall dramatically. The high concentration of NO_2_ could be due to dehydrogenation of ethanol, as suggested by Zabetta and Hupa, who revealed the formation of NO_2_ with a similar process but by using methanol [19]. The peak value of NO_2_ emission is just 15 ppm at 750°C when *α* = 0.6. The same trend is observed in figure 4*a*; the generation window of NO_2_ moves to a lower temperature as the addition increases.

Evolutions of NH_3_ slip are illustrated in figure 4*c*. The addition of ethanol additives has an obvious inhibitory effect on NH_3_ slip. NH_3_ slip increased without adding ethanol and fell by more than half because of the addition of ethanol during 700°C–800°C. When *α* = 0.6, the peak value of NH_3_ slip lowered to 8 ppm, which is almost one-tenth of the original level, thereby exhibiting a distinct improvement in NH_3_ slip. Serious NH_3_ slip at a low temperature may be due to insufficient active radicals at low temperatures, thereby hindering the conversion of NH_3_. Rapid oxidation of NH_3_ at a high temperature is the reason for the minimal NH_3_ slip within a large temperature window [14].

### 3.4. Effect of compound additives on the urea-based SNCR process

**Figure 5.**
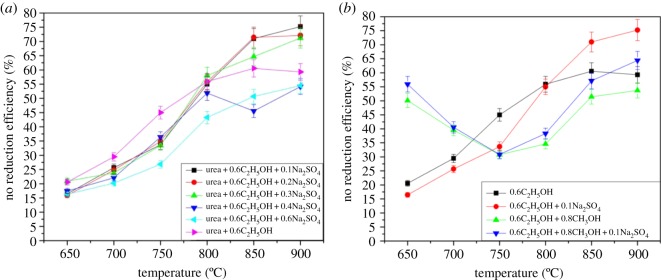
Effect of compound additives on the urea-based SNCR process.

Figure 5 presents the effect of compound additives and their molar concentration on NO reduction, with the compounds being C_2_H_5_OH, Na_2_SO_4_ and CH_3_OH. These additives are injected into the system by using a solution. Figure 5 shows that these additives have a certain effect on NO removal.

As shown in figure 5*a*, the little amount of Na_2_SO_4_ shows a positive result above 800°C, whereas the combination of Na_2_SO_4_ and C_2_H_5_OH has adverse effects on NO reduction below 800°C compared with the situation where only ethanol is the additive. Moreover, more Na_2_SO_4_ would lead to less denitration efficiency among the compound additives when Na_2_SO_4_/NO > 0.3, although Na species are investigated separately to have good performance in widening the temperature window and shifting the optimum reaction temperature to lower points [20]. The NO reduction efficiency is up to 70% and more when injecting 0.6C_2_H_5_OH + 0.1Na_2_SO_4_ additives (the number denotes the molar ratio of additive/NO) above 850°C, which is further improved on the basis of ethanol-only additive.

As shown in figure 5*b*, methanol promotes NO reduction significantly between 650°C and 725°C. Ethanol can be applied to the NO reduction above 725°C, while methanol is suitable for 650°C–725°C if adopted in compound additives. The NO reduction efficiency is above 55% at 650°C when adding 0.6C_2_H_5_OH + 0.8CH_3_OH + 0.1Na_2_SO_4_ additives. Na_2_SO_4_ improves NO reduction above 800°C in compound additives because of the reactions HOSO_2_ + O_2_ = HO_2_ + SO_3_ and NaSO_4_ + H_2_O = NaHSO_4_ + OH, which supply some HO_2_• and OH• [21]. This finding suggests that compound additives have a coupling effect on the SNCR process, and the coupling effect of different kinds of additives is critical to NO reduction and is worthy of further investigation, thereby providing a better choice for plants to reduce NO_X_ emissions.

### 3.5. Reaction mechanism analysis of urea-C_2_H_5_OH SNCR process

#### 3.5.1. Comparison of experimental data and simulated data

**Figure 6.**
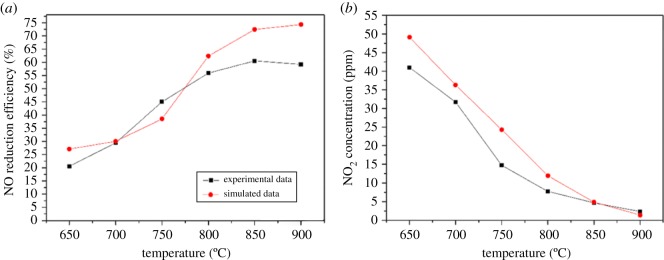
Comparison of experimental and simulated data.

Figure 6*a* shows a comparison of the NO reduction efficiency between experimental and simulated data. The simulated data agree well with the experimental data; the experimental efficiency ranges from 29.4 to 60%, whereas the simulated efficiency is 30–60.3%. Below 700°C, the simulation results are high because the SNCR reaction mechanisms are few under relatively low-temperature conditions, and the omission of related mechanism reactions leads to some errors, which is also acceptable. Above 800°C, the high simulation results are due to the idealized reaction conditions and the high reaction mixture in the simulation calculation.

Figure 6*b* shows that the overall trends of the simulated and experimental data are consistent when the temperature is higher than 700°C. The NO_2_ concentration exhibits a decreasing trend with the increase in temperature. The experimental data are subject to the analysis due to the relatively imperfect simulation mechanism at a low temperature. The peak of NO_2_ emission is 41 ppm at 650°C. At the beginning, NO_2_ is mainly generated through reaction (9). As the temperature increases, the NH_2_• and H• increase rapidly, thereby decreasing NO_2_ concentration via reactions (10) and (11).

$$\begin{array}{rl} & \mathrm{NO}+HO_{2}=NO_{2}+\mathrm{OH}\;\left(9\right)\\ & NO_{2}+NH_{2}=H_{2}\mathrm{NO}+\mathrm{NO}\;\left(10\right)\\ & NO_{2}+H=\mathrm{NO}+\mathrm{OH}\;\left(11\right) \end{array}$$

#### 3.5.2. Sensitivity analysis and pathways of NO

**Figure 7.**
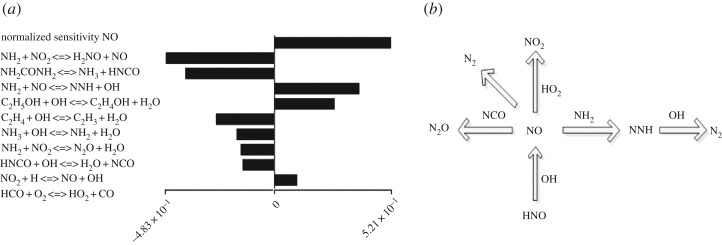
Sensitivity analysis and pathways of NO during urea-C_2_H_5_OH SNCR process.

The analysis results obtained at a temperature of 700°C are shown in figure 7*a*. The action of the NH_2_• is notable in the SNCR process. The competition between reactions (10) and (12) nearly determines the effect of the NH_2_•. The latter is dominant, which means that the NH_2_• is active in NO reduction; otherwise, it is favourable for NO production. Reactions (13), (15) and (16) predominate the NO removal at a low temperature.

As the temperature increases, reactions (12) and (14) play an important role in reducing NO. The addition of ethanol greatly promotes the formation of OH•, but reaction (17) dominates the formation of a large number of CO at a low temperature. Luckily, abundant OH• drives CO into CO_2_ as the temperature rises.

$$\begin{array}{rl} & NH_{2}+\mathrm{NO}=\mathrm{NNH}+\mathrm{OH}\;\left(12\right)\\ & NH_{2}\mathrm{CON}H_{2}=NH_{3}+\mathrm{HNCO}\;\left(13\right)\\ & NH_{3}+\mathrm{OH}=NH_{2}+H_{2}O\;\left(14\right)\\ & NH_{2}+NO_{2}=N_{2}O+H_{2}O\;\left(15\right)\\ & \mathrm{HNCO}+\mathrm{OH}=\mathrm{NCO}+H_{2}O\;\;\;\left(16\right)\\ & \mathrm{NCO}+NO_{2}=2\mathrm{NO}+\mathrm{CO}\;\left(17\right) \end{array}$$

The pathways of NO during the urea-C_2_H_5_OH-based SNCR process are illustrated in figure 7*b*. The major radicals are NCO•, NNH•, HNO•, HO_2_• and NH_2_•, which directly participate in the reduction and formation of NO. NCO may react with NO to create N_2_ and N_2_O; HNO• and OH• may be conducive to NO formation; and NH_2_• and NNH• can be used to convert NO to N_2_. Moreover, the HO_2_• and OH•, which are generated from ethanol decomposition and oxidation, limit NO_2_ emission and NH_3_ slip.

## 4. Conclusion

Ethanol additive can effectively shift the temperature window of the NO_X_OUT process to a lower range, and Na_2_SO_4_ additive is mixed with urea-C_2_H_5_OH solution to enhance NO reduction. The coupling effect of the compound additive is critical to NO removal. The detailed NO_X_OUT mechanism is explored through simulation and experimental study. The main conclusions are as follows:

(1) Adding ethanol to a urea solution provides potential ways for the SNCR NO_X_OUT process at a moderate temperature, and the NO_X_OUT efficiency ranges from 29% to 56% during 700–800°C.
(2) Sodium sulfate additive is active in NO reduction when injected into a urea-C_2_H_5_OH solution, thereby enhancing NO removal. This approach is a novel way to reduce NO_X_ emissions. Methanol additive is prone to improving the NO reduction below 725°C. A coupling effect exists between different kinds of additives for NO reduction; this effect deserves considerable attention and further research.
(3) New SNCR NO_X_OUT reaction routes are suggested for the urea-C_2_H_5_OH solution. Through a sensitivity analysis, the key elementary reactions are identified. According to the reaction routes, when ethanol is added to the urea solution at a proper rate, elementary reaction (HNCO + OH = H_2_O + NCO) is enhanced, thereby enabling ethanol decomposition to provide OH•, which works actively in reducing NO.

## Supplementary Material

Experimental configuration and simulation mechanism

## Supplementary Material

Certificate of English Editing

## Data accessibility

The datasets supporting this article have been uploaded as the electronic supplementary material.
