# Supplementary material for: Experiment and simulation study of the effect of ethanol and compound additives on the urea-based selective non-catalytic reduction process under moderate temperature conditions
Source: R Soc Open Sci. 2018 Oct 24;5(10):180969. doi: 10.1098/rsos.180969 (PMC6227956; doi:10.1098/rsos.180969)
Supplement: Experimental configuration and simulation mechanism [file rsos180969supp1.docx]

**Experimental Configuration:**

| Burner features | value | Concentration |
| --- | --- | --- |
| Temperature range(°C) | 650–900 |  |
| Total flow rate(L/min) | 2.5 |  |
| Inlet 2%NO(L/min) | 0.0513 | 410ppm |
| Inlet O_2_(L/min) | 0.075 | 3% |
| Inlet N_2_(L/min) | 2.3737(balance) |  |
| Injector features(Fig. 2) | value | Concentration |
| Total flow rate(ml/h) | 4.1 |  |
| NSR(urea) | 1 | 410ppm |
| NSR(ethanol) | 0–1 | 0–410ppm |
| Injector features(Fig. 3) | value | Concentration |
| Total flow rate(ml/h) | 4.1 |  |
| NSR(urea) | 1–1.5 | 410ppm–615ppm |
| NSR(ethanol) | 0.6 | 246ppm |
| Injector features(Fig. 4) | value | Concentration |
| Total flow rate(ml/h) | 4.1 |  |
| NSR(urea) | 1.25 | 512.5ppm |
| NSR(ethanol) | 0.6 | 246ppm |
| Injector features(Fig. 5(a)) | value | Concentration |
| Total flow rate(ml/h) | 10.3 |  |
| NSR(urea) | 1.25 | 512.5ppm |
| NSR(ethanol) | 0.6 | 246ppm |
| NSR(sodium sulfate) | 0–0.6 | 0–246ppm |
| Injector features(Fig. 5(b)) | value | Concentration |
| Total flow rate(ml/h) | 10.3 |  |
| NSR(urea) | 1.25 | 512.5ppm |
| NSR(ethanol) | 0.6 | 246ppm |
| NSR(sodium sulfate) | 0.1 | 41ppm |
| NSR(methanol) | 0.8 | 328ppm |

**Modelling Parameters**

|  | Ending Axial Position(cm) | Diameter(cm) | Temperature(°C) | Pressure(atm) |
| --- | --- | --- | --- | --- |
| value | 20 | 2.6 | 650–900 | 1.0 |

**Simulation Mechanism:**

| No. | Elementary reaction | A(cm^3^/mol s) | n | E(kj/mol) |
| --- | --- | --- | --- | --- |
| 1 | O+OH=H+O_2_ | 2.00E+14 | -0.4 | 0 |
| 2 | O+H_2_=OH+H | 5.06E+04 | 2.7 | 6290 |
| 3 | OH+H_2_=H_2_O+H | 2.14E+08 | 1.5 | 3450 |
| 4 | OH+OH=H_2_O+O | 4.33E+03 | 2.7 | -2486 |
| 5 | H+H+M=H_2_+M | 1.00E+18 | -1 | 0 |
| 6 | H+H+H_2_=H_2_+H_2_ | 9.20E+16 | -0.6 | 0 |
| 7 | H+H+H_2_O=H_2_+H_2_O | 6.00E+19 | -1.2 | 0 |
| 8 | H+O+M=OH+M | 6.20E+16 | -0.6 | 0 |
| 9 | H+OH+M=H_2_O+M | 8.40E+21 | -2 | 0 |
| 10 | O+O+M=O_2_+M | 1.90E+13 | 0 | -1788 |
| 11 | H_2_+O_2_=OH+OH | 1.70E+13 | 0 | 47780 |
| 12 | H+O_2_+M=HO_2_+M | 1.50E+12 | 0.6 | 0 |
| 13 | H+O_2_+N_2_=HO_2_+N_2_ | 6.70E+19 | -1.4 | 0 |
| 14 | HO_2_+H=H_2_+O_2_ | 4.28E+13 | 0 | 1411 |
| 15 | HO_2_+H=OH+OH | 1.69E+14 | 0 | 875 |
| 16 | HO_2_+H=O+H_2_O | 3.01E+13 | 0 | 1721 |
| 17 | HO_2_+O=OH+O_2_ | 3.25E+13 | 0 | 0 |
| 18 | HO_2_+OH=H_2_O+O_2_ | 2.90E+13 | 0 | -497 |
| 19 | HO_2_+HO_2_=H_2_O_2_+O_2_ | 1.30E+11 | 0 | -1630 |
| 20 | HO_2_+HO_2_=H_2_O_2_+O_2_ | 4.20E+14 | 0 | 11980 |
| 21 | H_2_O_2_+M=OH+OH+M | 1.30E+17 | 0 | 45500 |
| 22 | H_2_O_2_+H=HO_2_+H_2_ | 1.70E+12 | 0 | 3755 |
| 23 | H_2_O_2_+H=H_2_O+OH | 1.00E+13 | 0 | 3576 |
| 24 | H_2_O_2_+O=HO_2_+OH | 6.60E+11 | 0 | 3974 |
| 25 | H_2_O_2_+OH=H_2_O+HO_2_ | 7.80E+12 | 0 | 1330 |
| 26 | H_2_O_2_+OH=H_2_O+HO_2_ | 5.80E+14 | 0 | 9560 |
| 27 | CO+O+M=CO_2_+M | 6.20E+14 | 0 | 3000 |
| 28 | CO+OH=CO_2_+H | 1.50E+07 | 1.3 | -765 |
| 29 | CO+O_2_=CO_2_+O | 2.50E+12 | 0 | 47700 |
| 30 | CO+HO_2_=CO_2_+OH | 1.50E+14 | 0 | 23650 |
| 31 | H+NO+M=HNO+M | 4.00E+20 | -1.8 | 0 |
| 32 | NO+O+M=NO_2_+M | 7.50E+19 | -1.4 | 0 |
| 33 | OH+NO(+M)=HONO(+M) | 2.00E+12 | 0 | -721 |
| 34 | HO_2_+NO=NO_2_+OH | 2.11E+12 | 0 | -479 |
| 35 | NO_2_+H=NO+OH | 8.40E+13 | 0 | 0 |
| 36 | NO_2_+O=NO+O_2_ | 3.90E+12 | 0 | -238 |
| 37 | NO_2_+NO_2_=NO+NO+O_2_ | 1.63E+12 | 0 | 26123 |
| 38 | NO_2_+NO_2_=NO_3_+NO | 9.60E+09 | 0.7 | 20900 |
| 39 | NO_2_+O(+M)=NO_3_(+M) | 1.30E+13 | 0 | 0 |
| 40 | NO_3_+H=NO_2_+OH | 6.00E+13 | 0 | 0 |
| 41 | NO_3_+O=NO_2_+O_2_ | 1.00E+13 | 0 | 0 |
| 42 | NO_3_+OH=NO_2_+HO_2_ | 1.40E+13 | 0 | 0 |
| 43 | NO_3_+HO_2_=NO_2_+O_2_+OH | 1.50E+12 | 0 | 0 |
| 44 | NO_3_+NO_2_=NO+NO_2_+O_2_ | 4.90E+12 | 0 | 2940 |
| 45 | HNO+H=H_2_+NO | 4.46E+11 | 0.7 | 655 |
| 46 | HNO+O=NO+OH | 1.00E+13 | 0 | 0 |
| 47 | HNO+OH=NO+H_2_O | 3.60E+13 | 0 | 0 |
| 48 | HNO+O_2_=HO_2_+NO | 2.00E+12 | 0 | 25000 |
| 49 | HNO+NO_2_=HONO+NO | 6.00E+11 | 0 | 2000 |
| 50 | HNO+HNO=N_2_O+H_2_O | 9.00E+08 | 0 | 3100 |
| 51 | HNO+NH_2_=NH_3_+NO | 3.63E+06 | 1.6 | -1252 |
| 52 | H_2_NO+M=HNO+H+M | 2.80E+24 | -2.8 | 64915 |
| 53 | H_2_NO+M=HNOH+M | 1.10E+29 | -4 | 43982 |
| 54 | H_2_NO+H=HNO+H_2_ | 3.00E+07 | 2 | 2000 |
| 55 | H_2_NO+H=NH_2_+OH | 5.00E+13 | 0 | 0 |
| 56 | H_2_NO+O=HNO+OH | 3.00E+07 | 2 | 2000 |
| 57 | H_2_NO+O=NH_2_+O_2_ | 2.50E+14 | 0 | 0 |
| 58 | H_2_NO+OH=HNO+H_2_O | 2.00E+07 | 2 | 1000 |
| 59 | H_2_NO+NO=HNO+HNO | 2.00E+07 | 2 | 13000 |
| 60 | H_2_NO+NO_2_=HNO+HONO | 6.00E+11 | 0 | 2000 |
| 61 | H_2_NO+HO_2_=HNO+H_2_O_2_ | 2.90E+04 | 2.7 | -1600 |
| 62 | H_2_NO+O_2_=HNO+HO_2_ | 3.00E+12 | 0 | 25000 |
| 63 | HNOH+H=NH_2_+OH | 4.00E+13 | 0 | 0 |
| 64 | HNOH+H=HNO+H_2_ | 4.80E+08 | 1.5 | 378 |
| 65 | HNOH+O=HNO+OH | 7.00E+13 | 0 | 0 |
| 66 | HNOH+O=HNO+OH | 3.30E+08 | 1.5 | -358 |
| 67 | HNOH+OH=HNO+H_2_O | 2.40E+06 | 2 | -1192 |
| 68 | HNOH+NH_2_=NH_3_+HNO | 1.80E+06 | 1.9 | -1152 |
| 69 | HNOH+HO_2_=HNO+H_2_O_2_ | 2.90E+04 | 2.7 | -1600 |
| 70 | HNOH+M=HNO+H+M | 2.00E+24 | -2.8 | 58934 |
| 71 | HNOH+O_2_=HNO+HO_2_ | 3.00E+12 | 0 | 25000 |
| 72 | HNOH+NO_2_=HONO+HNO | 6.00E+11 | 0 | 2000 |
| 73 | HONO+H=H_2_+NO_2_ | 1.20E+13 | 0 | 7350 |
| 74 | HONO+O=OH+NO_2_ | 1.20E+13 | 0 | 6000 |
| 75 | HONO+OH=H_2_O+NO_2_ | 4.00E+12 | 0 | 0 |
| 76 | NH_3_+M=NH_2_+H+M | 2.20E+16 | 0 | 93470 |
| 77 | NH_3_+H=NH_2_+H_2_ | 6.36E+05 | 2.4 | 10171 |
| 78 | NH_3_+O=NH_2_+OH | 9.40E+06 | 1.9 | 6460 |
| 79 | NH_3_+OH=NH_2_+H_2_O | 2.04E+06 | 2 | 566 |
| 80 | NH_3_+HO_2_=NH_2_+H_2_O_2_ | 3.00E+11 | 0 | 22000 |
| 81 | NH_2_+H=NH+H_2_ | 4.00E+13 | 0 | 3650 |
| 82 | NH_2_+O=HNO+H | 6.63E+14 | -0.5 | 0 |
| 83 | NH_2_+O=NH+OH | 6.75E+12 | 0 | 0 |
| 84 | NH_2_+OH=NH+H_2_O | 4.00E+06 | 2 | 1000 |
| 85 | NH_2_+HO_2_=H_2_NO+OH | 5.00E+13 | 0 | 0 |
| 86 | NH_2_+HO_2_=NH_3_+O_2_ | 1.00E+13 | 0 | 0 |
| 87 | NH_2_+NO=NNH+OH | 2.29E+10 | 0.4 | -813.6 |
| 88 | NH_2_+NO_2_=N_2_O+H_2_O | 1.62E+16 | -1.4 | 268 |
| 89 | NH_2_+NO_2_=H_2_NO+NO | 6.48E+16 | -1.4 | 268 |
| 90 | NH_2_+H_2_NO=NH_3_+HNO | 3.00E+12 | 0 | 1000 |
| 91 | NH_2_+HONO=NO_2_+NH_3_ | 7.11E+01 | 3 | -4941 |
| 92 | NH_2_+NH_2_=N_2_H_2_+H_2_ | 8.50E+11 | 0 | 0 |
| 93 | NH_2_+NH=N_2_H_2_+H | 5.00E+13 | 0 | 0 |
| 94 | NH_2_+N=N_2_+H+H | 7.20E+13 | 0 | 0 |
| 95 | NH+H=N+H_2_ | 3.00E+13 | 0 | 0 |
| 96 | NH+O=NO+H | 9.20E+13 | 0 | 0 |
| 97 | NH+OH=HNO+H | 2.00E+13 | 0 | 0 |
| 98 | NH+OH=N+H_2_O | 5.00E+11 | 0.5 | 2000 |
| 99 | NH+O_2_=HNO+O | 4.61E+05 | 2 | 6500 |
| 100 | NH+O_2_=NO+OH | 1.28E+06 | 1.5 | 100 |
| 101 | NH+NO=N_2_O+H | 2.94E+14 | -0.4 | 0 |
| 102 | NH+NO=N_2_O+H | -2.20E+13 | -0.2 | 0 |
| 103 | NH+NO=N_2_+OH | 2.16E+13 | -0.2 | 0 |
| 104 | NH+NO_2_=N_2_O+OH | 1.00E+13 | 0 | 0 |
| 105 | NH+NH=N_2_+H+H | 2.54E+13 | 0 | 0 |
| 106 | NH+N=N_2_+H | 3.00E+13 | 0 | 0 |
| 107 | N+OH=NO+H | 3.80E+13 | 0 | 0 |
| 108 | N+O_2_=NO+O | 6.40E+09 | 1 | 6280 |
| 109 | N+NO=N_2_+O | 3.27E+12 | 0.3 | 0 |
| 110 | N_2_H_2_+M=NNH+H+M | 5.00E+16 | 0 | 50000 |
| 111 | N_2_H_2_+H=NNH+H_2_ | 5.00E+13 | 0 | 1000 |
| 112 | N_2_H_2_+O=NH_2_+NO | 1.00E+13 | 0 | 0 |
| 113 | N_2_H_2_+O=NNH+OH | 2.00E+13 | 0 | 1000 |
| 114 | N_2_H_2_+OH=NNH+H_2_O | 1.00E+13 | 0 | 1000 |
| 115 | N_2_H_2_+NO=N_2_O+NH_2_ | 3.00E+12 | 0 | 0 |
| 116 | N_2_H_2_+NH_2_=NH_3_+NNH | 1.00E+13 | 0 | 1000 |
| 117 | N_2_H_2_+NH=NNH+NH_2_ | 1.00E+13 | 0 | 1000 |
| 118 | NH_2_+NH_2_=NH_3_+NH | 5.00E+13 | 0 | 10000 |
| 119 | NH_2_+NH_2_(+M)=N_2_H_4_(+M) | 1.50E+13 | 0 | 0 |
| 120 | N_2_H_4_+H=N_2_H_3_+H_2_ | 1.30E+13 | 0 | 2500 |
| 121 | N_2_H_4_+O=N_2_H_2_+H_2_O | 8.50E+13 | 0 | 1200 |
| 122 | N_2_H_4_+OH=N_2_H_3_+H_2_O | 4.00E+13 | 0 | 0 |
| 123 | N_2_H_4_+NH_2_=N_2_H_3_+NH_3_ | 3.90E+12 | 0 | 1500 |
| 124 | N_2_H_3_+M=N_2_H_2_+H+M | 3.50E+16 | 0 | 46000 |
| 125 | N_2_H_3_+H=NH_2_+NH_2_ | 1.60E+12 | 0 | 0 |
| 126 | N_2_H_3_+O=N_2_H_2_+OH | 5.00E+12 | 0 | 5000 |
| 127 | N_2_H_3_+O=NH_2_+HNO | 1.00E+13 | 0 | 0 |
| 128 | N_2_H_3_+OH=N_2_H_2_+H_2_O | 1.00E+13 | 0 | 1000 |
| 129 | N_2_H_3_+OH=NH_3_+HNO | 1.00E+12 | 0 | 15000 |
| 130 | N_2_H_3_+NH=N_2_H_2_+NH_2_ | 2.00E+13 | 0 | 0 |
| 131 | HONO+HONO=NO+NO_2_+H_2_O | 2.30E+12 | 0 | 8400 |
| 132 | NNH=N_2_+H | 6.50E+07 | 0 | 0 |
| 133 | NNH+H=N_2_+H_2_ | 1.00E+14 | 0 | 0 |
| 134 | NNH+O=N_2_+OH | 8.00E+13 | 0 | 0 |
| 135 | NNH+O=N_2_O+H | 1.00E+14 | 0 | 0 |
| 136 | NNH+O=NH+NO | 5.00E+13 | 0 | 0 |
| 137 | NNH+OH=N_2_+H_2_O | 5.00E+13 | 0 | 0 |
| 138 | NNH+O_2_=N_2_+HO_2_ | 2.00E+14 | 0 | 0 |
| 139 | NNH+O_2_=N_2_+O_2_+H | 5.00E+13 | 0 | 0 |
| 140 | NNH+NO=N_2_+HNO | 5.00E+13 | 0 | 0 |
| 141 | NNH+NH_2_=N_2_+NH_3_ | 5.00E+13 | 0 | 0 |
| 142 | NNH+NH=N_2_+NH_2_ | 5.00E+13 | 0 | 0 |
| 143 | N_2_O+M=N_2_+O+M | 4.00E+14 | 0 | 56100 |
| 144 | N_2_O+H=N_2_+OH | 3.30E+10 | 0 | 4729 |
| 145 | N_2_O+H=N_2_+OH | 4.40E+14 | 0 | 19254 |
| 146 | N_2_O+O=NO+NO | 6.60E+13 | 0 | 26630 |
| 147 | N_2_O+O=N_2_+O_2_ | 1.00E+14 | 0 | 28000 |
| 148 | N_2_O+OH=N_2_+HO_2_ | 1.29E-02 | 4.7 | 36561 |
| 149 | N_2_O+OH=HNO+NO | 1.18E-04 | 4.3 | 25081 |
| 150 | N_2_O+NO=NO_2_+N_2_ | 5.26E+05 | 2.2 | 46281 |
| 151 | HCO+M=H+CO+M | 1.90E+17 | -1 | 17020 |
| 152 | HCO+H=H_2_+CO | 7.20E+13 | 0 | 0 |
| 153 | HCO+O=OH+CO | 3.00E+13 | 0 | 0 |
| 154 | HCO+O=H+CO_2_ | 3.00E+13 | 0 | 0 |
| 155 | HCO+OH=H_2_O+CO | 1.10E+14 | 0 | 0 |
| 156 | HCO+O_2_=HO_2_+CO | 7.60E+12 | 0 | 400 |
| 157 | HNCO+M=NH+CO+M | 1.10E+16 | 0 | 86000 |
| 158 | HNCO+H=NH_2_+CO | 2.25E+07 | 1.7 | 3800 |
| 159 | HNCO+O=NCO+OH | 2.20E+06 | 2.1 | 11430 |
| 160 | HNCO+O=NH+CO_2_ | 9.60E+07 | 1.4 | 8520 |
| 161 | HNCO+O=HNO+CO | 1.50E+08 | 1.6 | 44012 |
| 162 | HNCO+OH=H_2_O+NCO | 6.40E+05 | 2 | 2560 |
| 163 | HNCO+HO_2_=NCO+H_2_O_2_ | 3.00E+11 | 0 | 29000 |
| 164 | HNCO+O_2_=HNO+CO_2_ | 1.00E+12 | 0 | 35000 |
| 165 | HNCO+NH_2_=NH_3_+NCO | 5.00E+12 | 0 | 6200 |
| 166 | HNCO+NH=NH_2_+NCO | 3.00E+13 | 0 | 23700 |
| 167 | HNCO+NO_2_=HNNO+CO_2_ | 2.50E+12 | 0 | 26200 |
| 168 | NCO+M=N+CO+M | 3.10E+16 | -0.5 | 48000 |
| 169 | NCO+H=NH+CO | 5.00E+13 | 0 | 0 |
| 170 | NCO+O=NO+CO | 4.70E+13 | 0 | 0 |
| 171 | NCO+OH=NO+HCO | 5.00E+12 | 0 | 15000 |
| 172 | NCO+H_2_=HNCO+H | 7.60E+02 | 3 | 4000 |
| 173 | NCO+O_2_=NO+CO_2_ | 2.00E+12 | 0 | 20000 |
| 174 | NCO+HCO=HNCO+CO | 3.60E+13 | 0 | 0 |
| 175 | NCO+N=N_2_+CO | 2.00E+13 | 0 | 0 |
| 176 | NCO+NO=N_2_O+CO | 6.20E+17 | -1.7 | 763 |
| 177 | NCO+NO=N_2_+CO_2_ | 7.80E+17 | -1.7 | 763 |
| 178 | NCO+NO_2_=2NO+CO | 1.30E+13 | 0 | 0 |
| 179 | NCO+NO_2_=N_2_O+CO_2_ | 5.40E+12 | 0 | 0 |
| 180 | NCO+HNO=HNCO+NO | 1.80E+13 | 0 | 0 |
| 181 | NCO+HONO=HNCO+NO_2_ | 3.60E+12 | 0 | 0 |
| 182 | NCO+NCO=2CO+N_2_ | 1.80E+13 | 0 | 0 |
| 183 | NO+HCO=CO+HNO | 7.20E+12 | 0 | 0 |
| 184 | NO_2_+CO=CO_2_+NO | 9.00E+13 | 0 | 33800 |
| 185 | NO_2_+HCO=CO+HONO | 2.10E+00 | 3.3 | 2350 |
| 186 | NO_2_+HCO=H+CO_2_+NO | 8.40E+15 | -0.8 | 1930 |
| 187 | NH_2_CONH_2_=>NH_3_+HNCO | 1.27E+04 | 0 | 15540 |
| 188 | NH_2_CONH_2_+H_2_O=>2NH_3_+CO_2_ | 9.92E+03 | 0 | 20980 |
| 189 | H+O_2_(+N_2_)=HO_2_(+N_2_) | 4.52E+13 | 0 | 0 |
| 190 | H+O_2_(+H_2_)=HO_2_(+H_2_) | 4.52E+13 | 0 | 0 |
| 191 | H+O_2_(+H_2_O)=HO_2_(+H_2_O) | 4.52E+13 | 0 | 0 |
| 192 | HO_2_+HO_2_=H_2_O_2_+O_2_ | 4.20E+14 | 0 | 11982 |
| 193 | HO_2_+HO_2_=H_2_O_2_+O_2_ | 1.30E+11 | 0 | -1629 |
| 194 | CH_3_+CH_3_(+m)=C_2_H_6_(+m) | 9.22E+16 | -1.2 | 636 |
| 195 | CH_3_+H(+m)=CH_4_(+m) | 2.14E+15 | -0.4 | 0 |
| 196 | CH_4_+H=CH_3_+H_2_ | 2.20E+04 | 3 | 8750 |
| 197 | CH_4_+OH=CH_3_+H_2_O | 4.19E+06 | 2 | 2547 |
| 198 | CH_4_+O=CH_3_+OH | 6.92E+08 | 1.6 | 8485 |
| 199 | CH_4_+HO_2_=CH_3_+H_2_O_2_ | 1.12E+13 | 0 | 24640 |
| 200 | CH_3_+HO_2_=CH_3_O+OH | 7.00E+12 | 0 | 0 |
| 201 | CH_3_+HO_2_=CH_4_+O_2_ | 3.00E+12 | 0 | 0 |
| 202 | CH_3_+O=CH_2_O+H | 8.00E+13 | 0 | 0 |
| 203 | CH_3_+O_2_=CH_3_O+O | 1.45E+13 | 0 | 29209 |
| 204 | CH_3_+O_2_=CH_2_O+OH | 2.51E+11 | 0 | 14640 |
| 205 | CH_3_O+H=CH_3_+OH | 1.00E+13 | 0 | 0 |
| 206 | CH_2_OH+H=CH_3_+OH | 1.00E+13 | 0 | 0 |
| 207 | CH_3_+OH=CH_2_(s)+H_2_O | 2.00E+13 | 0 | 550 |
| 208 | CH_3_+OH=HCOH+H_2_ | 1.00E+10 | 0 | -415 |
| 209 | CH_3_+OH=CH_2_+H_2_O | 3.00E+06 | 2 | 2500 |
| 210 | CH_3_+H=CH_2_+H_2_ | 9.00E+13 | 0 | 15100 |
| 211 | CH_3_+m=CH+H_2_+m | 6.90E+14 | 0 | 82469 |
| 212 | CH_3_+m=CH_2_+H+m | 1.90E+16 | 0 | 91411 |
| 213 | CH_3_+OH(+m)=CH_3_OH(+m) | 8.70E+13 | 0.1 | 0 |
| 214 | CH_3_OH(+m)=CH_2_(s)+H_2_O(+m) | 2.84E+10 | 1 | 83871 |
| 215 | CH_3_OH(+m)=HCOH+H_2_(+m) | 4.20E+09 | 1.1 | 85604 |
| 216 | CH_3_OH(+m)=CH_2_O+H_2_(+m) | 2.03E+09 | 1 | 91443 |
| 217 | CH_3_OH+OH=CH_2_OH+H_2_O | 2.61E+05 | 2.2 | -1344 |
| 218 | CH_3_OH+OH=CH_3_O+H_2_O | 2.62E+06 | 2.1 | 916 |
| 219 | CH_3_OH+O=CH_2_OH+OH | 3.88E+05 | 2.5 | 3080 |
| 220 | CH_3_OH+H=CH_2_OH+H_2_ | 1.70E+07 | 2.1 | 4868 |
| 221 | CH_3_OH+H=CH_3_O+H_2_ | 4.24E+06 | 2.1 | 4868 |
| 222 | CH_3_OH+CH_3_=CH_2_OH+CH_4_ | 3.19E+01 | 3.2 | 7171 |
| 223 | CH_3_OH+CH_3_=CH_3_O+CH_4_ | 1.45E+01 | 3.1 | 6935 |
| 224 | CH_3_OH+HO_2_=CH_2_OH+H_2_O_2_ | 9.64E+10 | 0 | 12578 |
| 225 | CH_2_O+H(+m)=CH_3_O(+m) | 5.40E+11 | 0.5 | 2600 |
| 226 | CH_2_O+H(+m)=CH_2_OH(+m) | 5.40E+11 | 0.5 | 3600 |
| 227 | CH_3_O+CH_3_=CH_2_O+CH_4_ | 1.20E+13 | 0 | 0 |
| 228 | CH_3_O+O=CH_2_O+OH | 1.00E+13 | 0 | 0 |
| 229 | CH_2_OH+O=CH_2_O+OH | 1.00E+13 | 0 | 0 |
| 230 | CH_3_O+O_2_=CH_2_O+HO_2_ | 6.30E+10 | 0 | 2600 |
| 231 | CH_2_OH+O_2_=CH_2_O+HO_2_ | 1.57E+15 | -1 | 0 |
| 232 | CH_2_OH+O_2_=CH_2_O+HO_2_ | 7.23E+13 | 0 | 3577 |
| 233 | HCOH+OH=HCO+H_2_O | 2.00E+13 | 0 | 0 |
| 234 | HCOH+O=CO+OH+H | 8.00E+13 | 0 | 0 |
| 235 | HCOH+O_2_=CO+OH+OH | 1.00E+13 | 0 | 0 |
| 236 | CH_2_+O=CO+H+H | 5.00E+13 | 0 | 0 |
| 237 | CH_2_+O=CO+H_2_ | 3.00E+13 | 0 | 0 |
| 238 | CH_2_+O_2_=CH_2_O+O | 3.29E+21 | -3.3 | 2868 |
| 239 | CH_2_+O_2_=CO_2_+H+H | 3.29E+21 | -3.3 | 2868 |
| 240 | CH_2_+O_2_=CO_2_+H_2_ | 1.01E+21 | -3.3 | 1508 |
| 241 | CH_2_+O_2_=CO+H_2_O | 7.28E+19 | -2.5 | 1809 |
| 242 | CH_2_+O_2_=HCO+OH | 1.29E+20 | -3.3 | 284 |
| 243 | CH_2_+CH_3_=C_2_H_4_+H | 4.00E+13 | 0 | 0 |
| 244 | CH_2_+CH_2_=C_2_H_2_+H+H | 4.00E+13 | 0 | 0 |
| 245 | CH_2_+HCCO=C_2_H_3_+CO | 3.00E+13 | 0 | 0 |
| 246 | CH_2_+C_2_H_2_=H_2_CCCH+H | 1.20E+13 | 0 | 6600 |
| 247 | CH_2_(s)+m=CH_2_+m | 1.00E+13 | 0 | 0 |
| 248 | CH_2_(s)+CH_4_=CH_3_+CH_3_ | 4.00E+13 | 0 | 0 |
| 249 | CH_2_(s)+C_2_H_6_=CH_3_+C_2_H_5_ | 1.20E+14 | 0 | 0 |
| 250 | CH_2_(s)+O_2_=CO+OH+H | 7.00E+13 | 0 | 0 |
| 251 | CH_2_(s)+H_2_=CH_3_+H | 7.00E+13 | 0 | 0 |
| 252 | CH_2_(s)+C_2_H_2_=H_2_CCCH+H | 1.50E+14 | 0 | 0 |
| 253 | CH_2_(s)+C_2_H_4_=aC_3_H_5_+H | 1.30E+14 | 0 | 0 |
| 254 | CH_2_(s)+O=CO+H+H | 3.00E+13 | 0 | 0 |
| 255 | CH_2_(s)+CH_3_=C_2_H_4_+H | 2.00E+13 | 0 | 0 |
| 256 | CH+O=CO+H | 5.70E+13 | 0 | 0 |
| 257 | CH+H_2_O=CH_2_O+H | 1.17E+15 | -0.8 | 0 |
| 258 | CH+CH_2_O=CH_2_CO+H | 9.46E+13 | 0 | -515 |
| 259 | CH+C_2_H_2_=C_3_H_2_+H | 1.00E+14 | 0 | 0 |
| 260 | CH+CH_2_=C_2_H_2_+H | 4.00E+13 | 0 | 0 |
| 261 | CH+CH_3_=C_2_H_3_+H | 3.00E+13 | 0 | 0 |
| 262 | CH+CH_4_=C_2_H_4_+H | 6.00E+13 | 0 | 0 |
| 263 | HCOOH+CH_3_=CH_4_+CO+OH | 3.90E-07 | 5.8 | 2200 |
| 264 | HCOOH+O=CO+OH+OH | 1.77E+18 | -1.9 | 2975 |
| 265 | CH_2_O+OH=HCO+H_2_O | 3.43E+09 | 1.2 | -447 |
| 266 | CH_2_O+H=HCO+H_2_ | 2.19E+08 | 1.8 | 3000 |
| 267 | CH_2_O+m=HCO+H+m | 3.31E+16 | 0 | 81000 |
| 268 | CH_2_O+O=HCO+OH | 1.80E+13 | 0 | 3080 |
| 269 | C_2_H_5_OH(+m)=CH_3_+CH_2_OH(+m) | 5.94E+23 | -1.7 | 91163 |
| 270 | C_2_H_5_OH(+m)=C_2_H_5_+OH(+m) | 1.25E+23 | -1.5 | 96005 |
| 271 | C_2_H_5_OH(+m)=C_2_H_4_+H_2_O(+m) | 2.79E+13 | 0.1 | 66136 |
| 272 | C_2_H_5_OH(+m)=CH_3_HCO+H_2_(+m) | 7.24E+11 | 0.1 | 91007 |
| 273 | C_2_H_5_OH+OH=C_2_H_4_OH+H_2_O | 1.74E+11 | 0.3 | 600 |
| 274 | C_2_H_5_OH+H=C_2_H_4_OH+H_2_ | 1.23E+07 | 1.8 | 5098 |
| 275 | C_2_H_5_OH+O=C_2_H_4_OH+OH | 9.41E+07 | 1.7 | 5459 |
| 276 | C_2_H_5_OH+CH_3_=C_2_H_4_OH+CH_4_ | 2.19E+02 | 3.2 | 9622 |
| 277 | C_2_H_5_OH+HO_2_=CH_3_CHOH+H_2_O_2_ | 8.20E+03 | 2.5 | 10750 |
| 278 | CH_3_CH_2_O+m=CH_3_HCO+H+m | 1.16E+35 | -5.9 | 25274 |
| 279 | CH_3_CHOH+O=CH_3_HCO+OH | 1.00E+14 | 0 | 0 |
| 280 | CH_3_CHOH+HO_2_=CH_3_HCO+OH+OH | 4.00E+13 | 0 | 0 |
| 281 | CH_3_HCO+OH=CH_3_+HCOOH | 3.00E+15 | -1.1 | 0 |
| 282 | CH_3_HCO+O_2_=CH_3_CO+HO_2_ | 1.00E+14 | 0 | 42200 |
| 283 | C_2_H_6_+O=C_2_H_5_+OH | 3.00E+07 | 2 | 5115 |
| 284 | C_2_H_6_+OH=C_2_H_5_+H_2_O | 7.23E+06 | 2 | 864 |
| 285 | C_2_H_5_+H=C_2_H_4_+H_2_ | 1.25E+14 | 0 | 8000 |
| 286 | C_2_H_5_+H=CH_3_+CH_3_ | 3.00E+13 | 0 | 0 |
| 287 | C_2_H_5_+H=C_2_H_6_ | 3.00E+13 | 0 | 0 |
| 288 | C_2_H_5_+OH=C_2_H_4_+H_2_O | 4.00E+13 | 0 | 0 |
| 289 | C_2_H_5_+O=CH_3_+CH_2_O | 1.00E+14 | 0 | 0 |
| 290 | C_2_H_5_+HO_2_=C_2_H_6_+O_2_ | 3.00E+12 | 0 | 0 |
| 291 | C_2_H_5_+HO_2_=CH_3_CH_2_O+OH | 3.00E+13 | 0 | 0 |
| 292 | C_2_H_5_+O_2_=C_2_H_4_+HO_2_ | 2.89E+28 | -5.4 | 7585 |
| 293 | C_2_H_5_+O_2_=CH_3_HCO+OH | 4.90E+11 | -0.5 | 8357 |
| 294 | C_2_H_4_+OH=C_2_H_4_OH | 1.29E+12 | 0 | -817 |
| 295 | C_2_H_4_OH+O_2_=HOC_2_H_4_O_2_ | 1.00E+12 | 0 | -1100 |
| 296 | HOC_2_H_4_O_2_=CH_2_O+CH_2_O+OH | 6.00E+10 | 0 | 24500 |
| 297 | C_2_H_4_+OH=C_2_H_3_+H_2_O | 2.02E+13 | 0 | 5936 |
| 298 | C_2_H_4_+O=CH_3_+HCO | 1.02E+07 | 1.9 | 179 |
| 299 | C_2_H_4_+O=CH_2_HCO+H | 3.39E+06 | 1.9 | 179 |
| 300 | C_2_H_4_+CH_3_=C_2_H_3_+CH_4_ | 6.62E+00 | 3.7 | 9500 |
| 301 | C_2_H_4_+H=C_2_H_3_+H_2_ | 3.36E-07 | 6 | 1692 |
| 302 | C_2_H_4_+H(+m)=C_2_H_5_(+m) | 1.08E+12 | 0.5 | 1822 |
| 303 | C_2_H_4_(+m)=C_2_H_2_+H_2_(+m) | 1.80E+14 | 0 | 87000 |
| 304 | C_2_H_3_+H(+m)=C_2_H_4_(+m) | 6.10E+12 | 0.3 | 280 |
| 305 | C_2_H_3_+H=C_2_H_2_+H_2_ | 9.00E+13 | 0 | 0 |
| 306 | C_2_H_3_+O=CH_2_CO+H | 3.00E+13 | 0 | 0 |
| 307 | C_2_H_3_+O_2_=CH_2_O+HCO | 1.70E+29 | -5.3 | 6500 |
| 308 | C_2_H3+O_2_=CH_2_HCO+O | 5.50E+14 | -0.6 | 5260 |
| 309 | C_2_H_3_+O_2_=C_2_H_2_+HO_2_ | 2.12E-06 | 6 | 9484 |
| 310 | C_2_H_3_+OH=C_2_H_2_+H_2_O | 2.00E+13 | 0 | 0 |
| 311 | C_2_H_3_+C_2_H=C_2_H_2_+C_2_H_2_ | 3.00E+13 | 0 | 0 |
| 312 | C_2_H_3_+CH=CH_2_+C_2_H_2_ | 5.00E+13 | 0 | 0 |
| 313 | C_2_H_3_+CH_3_=aC_3_H_5_+H | 4.73E+02 | 3.7 | 5677 |
| 314 | C_2_H_3_+CH_3_=C_3_H_6_ | 4.46E+56 | -13 | 13865 |
| 315 | C_2_H_3_+CH_3_=C_2_H_2_+CH_4_ | 2.00E+13 | 0 | 0 |
| 316 | C_2_H_2_+OH=C_2_H+H_2_O | 3.37E+07 | 2 | 14000 |
| 317 | C_2_H_2_+OH=HCCOH+H | 5.04E+05 | 2.3 | 13500 |
| 318 | C_2_H_2_+OH=CH_2_CO+H | 2.18E-04 | 4.5 | -1000 |
| 319 | C_2_H_2_+OH=CH_2_CO+H | 2.00E+11 | 0 | 0 |
| 320 | C_2_H_2_+OH=CH_3_+CO | 4.83E-04 | 4 | -2000 |
| 321 | HCCOH+H=CH_2_CO+H | 1.00E+13 | 0 | 0 |
| 322 | C_2_H_2_+O=CH_2_+CO | 6.12E+06 | 2 | 1900 |
| 323 | C_2_H_2_+O=HCCO+H | 1.43E+07 | 2 | 1900 |
| 324 | C_2_H_2_+O=C_2_H+OH | 3.16E+15 | -0.6 | 15000 |
| 325 | C_2_H_2_+CH_3_=C_2_H+CH_4_ | 1.81E+11 | 0 | 17289 |
| 326 | C_2_H_2_+O_2_=HCCO+OH | 4.00E+07 | 1.5 | 30100 |
| 327 | C_2_H_2_+m=C_2_H+H+m | 4.20E+16 | 0 | 107000 |
| 328 | C_2_H_2_+H(+m)=C_2_H_3_(+m) | 3.11E+11 | 0.6 | 2589 |
| 329 | CH_2_HCO+H=CH_3_+HCO | 5.00E+13 | 0 | 0 |
| 330 | CH_2_HCO+H=CH_2_CO+H_2_ | 2.00E+13 | 0 | 0 |
| 331 | CH_2_HCO+O=CH_2_O+HCO | 1.00E+14 | 0 | 0 |
| 332 | CH_2_HCO+OH=CH_2_CO+H_2_O | 3.00E+13 | 0 | 0 |
| 333 | CH_2_HCO+O_2_=CH_2_O+CO+OH | 3.00E+10 | 0 | 0 |
| 334 | CH_2_HCO+CH_3_=C_2_H_5_+CO+H | 4.90E+14 | -0.5 | 0 |
| 335 | CH_2_HCO+HO_2_=CH_2_O+HCO+OH | 7.00E+12 | 0 | 0 |
| 336 | CH_2_HCO+HO_2_=CH_3_HCO+O_2_ | 3.00E+12 | 0 | 0 |
| 337 | CH_2_HCO=CH_3_+CO | 1.17E+43 | -9.8 | 43756 |
| 338 | CH_2_HCO=CH_2_CO+H | 1.81E+43 | -9.6 | 45868 |
| 339 | CHOCHO(+m)=CH_2_O+CO(+m) | 4.27E+12 | 0 | 50600 |
| 340 | CHOCHO+O=HCO+CO+OH | 7.24E+12 | 0 | 1970 |
| 341 | CH_2_CO+H=CH_3_+CO | 2.71E+04 | 2.8 | 714 |
| 342 | CH_2_CO+H=HCCO+H_2_ | 2.00E+14 | 0 | 8000 |
| 343 | CH_2_CO+O=HCCO+OH | 1.00E+13 | 0 | 8000 |
| 344 | CH_2_CO+OH=HCCO+H_2_O | 1.00E+13 | 0 | 2000 |
| 345 | CH_2_CO+OH=CH_2_OH+CO | 3.73E+12 | 0 | -1013 |
| 346 | CH_2_CO(+m)=CH_2_+CO(+m) | 3.00E+14 | 0 | 70980 |
| 347 | C_2_H+H_2_=C_2_H_2_+H | 4.09E+05 | 2.4 | 864.3 |
| 348 | C_2_H+O_2_=CO+CO+H | 9.04E+12 | 0 | -457 |
| 349 | HCCO+O=H+CO+CO | 8.00E+13 | 0 | 0 |
| 350 | HCCO+O_2_=HCO+CO+O | 2.50E+08 | 1 | 0 |
| 351 | HCCO+O_2_=CO_2_+HCO | 2.40E+11 | 0 | -854 |
| 352 | HCCO+CH=C_2_H_2_+CO | 5.00E+13 | 0 | 0 |
| 353 | C_3_H_8_+O=NC_3_H_7_+OH | 3.73E+06 | 2.4 | 5504 |
| 354 | C_3_H_8_+O=iC_3_H_7_+OH | 5.48E+05 | 2.5 | 3139 |
| 355 | C_3_H_6_+O=CH_3_CHCO+H+H | 5.01E+07 | 1.8 | 76 |
| 356 | C_3_H_6_+O=C_2_H_5_+HCO | 1.58E+07 | 1.8 | -1216 |
| 357 | C_3_H_6_+O=aC_3_H_5_+OH | 5.24E+11 | 0.7 | 5884 |
| 358 | C_3_H_6_+H=C_2_H_4_+CH_3_ | 7.23E+12 | 0 | 1302 |
| 359 | C_3_H_6_+H=aC_3_H_5_+H_2_ | 1.73E+05 | 2.5 | 2492 |
| 360 | aC_3_H_5_+HO_2_=C_3_H_6_+O_2_ | 3.00E+12 | 0 | 0 |
| 361 | C_3_H_6_+CH_3_=aC_3_H_5_+CH_4_ | 2.22E+00 | 3.5 | 5675 |
| 362 | CH_3_CHCO+O=CH_3_+HCO+CO | 3.00E+07 | 2 | 0 |
| 363 | CH_2_CHCHO+O=CH_2_CO+HCO+H | 5.01E+07 | 1.8 | 76 |
| 364 | aC_3_H_5_+O_2_=CH_2_CHCHO+OH | 1.82E+13 | -0.4 | 22859 |
| 365 | aC_3_H_5_+O_2_=aC_3_H_4_+HO_2_ | 4.99E+15 | -1.4 | 22428 |
| 366 | aC_3_H_5_+O_2_=C_2_H_2_+CH_2_O+OH | 2.78E+25 | -4.8 | 15468 |
| 367 | aC_3_H_5_+HO_2_=CH_2_CHCH_2_O+OH | 1.00E+13 | 0 | 0 |
| 368 | CH_2_CHCH_2_O+O_2_=CH_2_CHCHO+HO_2_ | 4.00E+10 | 0 | 1100 |
| 369 | CH_2_CHCH_2_O+CO=aC_3_H_5_+CO_2_ | 4.68E+02 | 3.2 | 5380 |
| 370 | aC_3_H_5_+H=aC_3_H_4_+H_2_ | 5.00E+13 | 0 | 0 |
| 371 | aC_3_H_5_+O=CH_2_CHCHO+H | 1.81E+14 | 0 | 0 |
| 372 | pC_3_H_5_+O_2_=CH_3_CHCO+H+O | 1.60E+15 | -0.8 | 3135 |
| 373 | pC_3_H_5_+O=CH_3_CHCO+H | 1.00E+14 | 0 | 0 |
| 374 | C_3_H_2_+O_2_=HCCO+CO+H | 2.00E+12 | 0 | 1000 |

The kinetics of those reactions are expressed as: $k=A\left( T/{T_{0}} \right)^{n}e^{-E/{RT}}$, where k is the reaction rate constant; T is the absolute temperature; A is the pre-exponential factor; E is the activation energy; R is the universal gas contant.
