# Supplementary material for: Experiment and simulation study of the effect of ethanol and compound additives on the urea-based selective non-catalytic reduction process under moderate temperature conditions
Source: R Soc Open Sci. 2018 Oct 24;5(10):180969. doi: 10.1098/rsos.180969 (PMC6227956; doi:10.1098/rsos.180969)
Supplement: Certificate of English Editing [file rsos180969supp2.pdf]

ShineWrite.com
Professional Editing Support

Certificate of English Editing

To whom it may concern:

This memo certifies that one of our clients had contracted our academic editing service for the following file (s).

Order Number:
40-2018061423175691

Word Count:
4250 ~ 4675 words

Date of the review:
6/14/2018 (MM/DD/YYYY)

The English review was conducted using a two-stage process in which a junior editor first reviewed the file, and then a senior editor conducted the final and more thorough review. All our editors are native English speakers.

We would like to emphasize that our service is targeted on grammar and language edits. We do not rewrite the documents from scratch. If you feel certain points are incorrect, please call our attention by contacting service@shinewrite.com

Documents receiving this certification should be English-ready for publication - however, the author has the ability to accept or reject our suggestions and changes.

John J.

John J

Editor in charge
